# Research Needed to Support Clinical Use of Biomarkers as Prognostic Indicators for Patients with Heart Failure

**DOI:** 10.4061/2010/453851

**Published:** 2010-06-06

**Authors:** Thomas S. Rector, Inder S. Anand

**Affiliations:** ^1^Center for Chronic Disease Outcomes Research, VA Medical Center, One Veterans Drive, Minneapolis, MN 55417, USA; ^2^Department of Medicine, University of Minnesota, Minneapolis, MN 55455, USA; ^3^Department of Cardiology, VA Medical Center, One Veterans Drive, Minneapolis, MN 55417, USA

## Abstract

Despite extensive research and numerous publications biomarkers have yet to fulfill their promise as prognostic indicators that can be widely used in the care of patients with heart failure. Specific clinical applications need to be identified for informative analyses of data that emphasize the most directly applicable measures of predictive performance.

## 1. Introduction

A number of secondary analyses of data gathered during clinical trials and observational cohort studies have identified several biomarkers that have been touted as “powerful predictors” of morbid events and mortality in patients with heart failure [1, 2]. The resources that are being devoted to study molecular pathophysiology and genetics most likely will lead to identification of additional biomarkers that will provide prognostic information. In an era of evidence-based medicine when implementation of the results of funded research is very important, there has been a remarkable paucity of studies to make available forthright evidence that supports the clinical use of biomarkers as prognostic indicators in the care of patients with heart failure. Most published reports concerning the prognostic information provided by biomarkers including our own have not gone beyond merely using regression analyses to report associations between biomarkers and patient outcomes and posthoc discrimination of outcomes that are not directly applicable to prospective predictions needed for clinical practice. To support the clinical use of biomarkers as prognostic variables, investigators need to propose specific clinical applications, report more pertinent statistical analyses such as predictive values, and study patients typically encountered in clinical practice.

## 2. What Is the Use of Prognostic Markers for Care of Patients with Heart Failure?

A prognostic indicator (predictor) can be any variable or combination of variables that is measured to make probabilistic predictions about whether a defined clinical outcome will or will not occur. This definition includes risk factors that might be used to estimate the risk of developing heart failure and prognostic indicators that might be used to help determine the prognosis of patients who have an established diagnosis. Although studies of the use of biomarkers such as brain natriuretic peptide (BNP) to guide treatment decisions based on the pathophysiology of heart failure have addressed a very important potential clinical use of biomarkers, studies have not focused on the clinical use of biomarkers as prognostic variables [3, 4]. To conduct practical studies of biomarkers as prognostic indicators we need to focus on specific decisions encountered in practice or by policymakers that depend, in part, on a prognostic or risk assessment. Heart failure guidelines do employ prognostic factors to recommend who should be treated with medications and devices [5]. For example, recommendations for cardiac resynchronization therapy are based, in part, on a patient's ejection fraction, QRS interval, and NYHA class. However, these recommendations are driven primarily by the characteristics of the patients that were included in clinical trials that demonstrated efficacy rather than an explicit risk or prognostic assessment. A decision framework that places patients into different prognostic or risk groups who would be treated differently is needed to facilitate clinical studies of biomarkers as useful prognostic indicators. For example, some have reasonably suggested that measurement of BNP before discharging patients from hospital care for heart failure might be useful to identify those who have a small chance of an early readmission and would not need more intense follow-up or disease management [6]. Conversely, health care providers might want to identify patients with heart failure whose level of risk of subsequent hospital admission warrants closer medical monitoring. Bettencourt et al. reported that N-terminal pro-BNP was associated with hospitalization-free survival after patients were discharged from a hospital admission for heart failure [7]. They presented hospitalization-free survival curves for prognostic groups defined by the median N-terminal pro-BNP. Below the median value in their sample the event rate was a little over 20% after 6 months. The important question clinicians need to address is whether this cumulative rate of readmission or death is low enough to forego closer postdischarge follow-up? How low would the risk of readmission have to be before most health care providers would be willing to forego closer follow-up, and what level of N-terminal pro-BNP can predict the clinically acceptable level of risk? To date, most studies of prognostic biomarkers have not been designed or analyzed to address these types of clinically important questions. One recent study did suggest how a multivariable prediction model for mortality could be used to identify patients who most likely would not benefit from an implantable cardioverter-defibrillator [8]. 

In practice, clinicians implicitly classify each individual patient into a dichotomy of those that have a prognosis or level of risk that in the practitioner's judgment either does or does not warrant use of a medical intervention. The dichotomy is often thought about in relative qualitative terms such as “high risk” or “higher risk” rather than estimates of the probability that an outcome will or will not occur within a specified period of time. The outcome probability thresholds that help define clinical decision dichotomies need to be made more explicit to design and analyze studies that seek to determine whether measurement of one or more biomarkers substantially improves the ability of health care providers to classify patients into different risk or prognostic groups. We need to elicit a clinical consensus for outcome probability thresholds for the proposed clinical uses of a biomarker. Alternatively, investigators could propose and justify a decision threshold. Most likely these thresholds will be a bit fuzzy and there may be a range of equivocal outcome probabilities. Ideally the thresholds should take into account the net benefit, risk, and costs of interventions that would or would not be used in different prognostic groups. Nevertheless, pivotal studies of the use of prognostic biomarkers need to describe the clinical scenario for the proposed use and the prognostic or risk groups that would be treated differently. In other words, what does a patient's risk of adverse outcome have to be before a specific type of medical care is offered or withheld? If the answer is that any level of risk generally warrants medical intervention, then there is no need for biomarkers as prognostic predictors. For example, angiotensin converting enzyme inhibitors are generally recommended for patients with a left ventricular ejection fraction less than 35% irrespective of their absolute level of risk of adverse clinical outcomes [5].

## 3. What Is the Most Pertinent Measureof Predictive Performance?

Studies of prognostic indicators typically report a variety of statistical analyses including some newer methods such as reclassification tables [9, 10]. Most of these statistics are not directly applicable to clinical practice. The clinician's or policymaker's objective is usually to place patients into groups that would be treated differently recognizing that there will be substantial prediction errors for individual patients in each group [11, 12]. Currently use of prognostic indicators in the care of heart failure is largely limited to qualitative characterization of patients enrolled in clinical trials rather than to making quantitative outcome predictions. However, prognostic biomarkers could be used to select patients for clinical trails in a manner that is more explicitly based on predicted outcome probabilities. Currently, the mortality rate in many cohorts of study-eligible patients with heart failure is too low to be able to detect a survival benefit without a prohibitively large and costly study. Whether one or more prognostic indicators can help identify patients who have a “high-enough mortality risk” is an important question [13]. To answer this question, one first has to define “high risk” according to how patients would be selected for the treatment in practice. For the sake of discussion, let us say we want to treat patients who have at least a 10% estimated probability of dying within one year. 

Typically, clinical trials like the Valsartan Heart Failure Trial (Val-HeFT) include baseline assessments of several recognized prognostic indicators such as age, NYHA class, left ventricular ejection fraction, systolic blood pressure, renal function (serum creatinine, estimated glomerular filtration rate, blood urea nitrogen, and presence of proteinuria), serum sodium and albumin concentrations, hemoglobin, comorbidities including diabetes and atrial fibrillation, and current treatments [14]. If we were to use these available assessments to somehow estimate each patient's 1-year mortality risk and select a group whose predicted risk during the first year is at least 10%, the most relevant measure of our prognostic performance would be whether the *observed* 1-year mortality in the selected group was indeed at least 10%. This could be determined by estimating the positive predictive value (PPV), the observed percentage of subjects placed in the “higher-risk” group who die within one year. The negative predictive value (NPV), the percentage of excluded “lower-risk” subjects who do not die within one year, might be of interest as well. 

After reviewing the extensive literature about the prognostic information provided by the previously listed baseline variables in the Val-HeFT data base, we were not very confident in our ability to select study-eligible patients who would have at least a 10% risk of dying within 1 year. Therefore, baseline variables collected in Val-HeFT were analyzed to help us select patients based on their predicted probability of dying within the first year of follow-up. We did not try to optimize the prediction model that is merely being used as example and only included the subjects who had data for all variables including the biomarkers that are discussed herein (*n* = 3,551 out of the 5,010 in the Val-HeFT study). Consistent with many previous studies, several of the baseline variables (age, NYHA class, systolic blood pressure, ejection fraction, serum sodium, glomerular filtration rate, proteinuria and hemoglobin) were statistically significant in a multivariable logistic regression model of the probability of dying within a year. Given the estimated regression coefficients, we entered each patient's baseline values for the predictors into the logistic regression equation to calculate each subject's predicted probability of dying within the first year. The PPV in the group that had a predicted probability of dying of at least 0.1(10%) was 16.6% (200 deaths out of 1206 subjects). Thus, we could use these clinical variables to select a subset of patients among whom at least 10% would be expected to die during the first year assuming the prediction model would be valid for future patients as well. Overall, use of the prediction model would enroll 34% (1206/3551) of the subjects screened; however, those enrolled would have higher expected first-year mortality (16.6%) than the entire screened group (9.3%). Use of the predictive model would exclude the remaining 66% of screened patients from enrollment (treatment). The NPV was 94.4% (2214 survivors out of 2345 subjects were classified as <10% risk). Thus, 5.6% of the patients who would be excluded from a trial based on this prediction model would be expected to die. 

Clearly the estimated predictive values will change as follow-up continues and more patients experience the outcome event being predicted or are lost to follow-up. The PPV in a patient group classified as having ≥10% mortality during the first year can be summarized using time-to-event plots, for example, plots of the cumulative mortality. The NPV in the patient groups classified as having ≤10% mortality during the first year can be summarized using Kaplan-Meier plots of cumulative survival.

Less clinically relevant to assessing the predictive model's performance, the estimated sensitivity of the predictive model was 60% at the cutoff for predicted probabilities of ≥0.1 (10%). Thus, if we elected to enroll the 34% (1206/3551) of study-eligible subjects with ≥10% predicted probability of dying *and looked back* after one-year elapsed we would expect to have included 60% of the subjects who died. The estimated specificity was 69%. Use of this predictive model would be expected to exclude 69% who would not die.

## 4. How Much Might Newer Biomarkers Improve Predictive Performance?

Several previous reports suggested that biomarkers including BNP and serum troponin T measured by a highly sensitive assay (hs-TnT) are associated with all-cause mortality [10, 15, 16]. The median (interquartile range) for the baseline BNP levels in the Val-HeFT sample was 109 (45 to 254 pg/mL) and 13.7 (7.3 to 23.4 ng/mL) for hs-TnT. What's the most informative way to determine whether addition of these biomarkers to the baseline clinical prediction model would improve the predictive performance? 

### 4.1. Measures of Association

When the natural logarithmic transformations of both BNP and hs-TnT levels were added to the previous baseline clinical prediction model, both were independently associated with 1-year mortality (odds ratio for BNP = 1.4 with 95% confidence interval 1.2 to 1.5; *P* < .0001 and hs-TnT = 1.7 with 95% confidence interval 1.4 to 2.0; *P* < .0001). However, finding a statistically significant independent association (in this example a statistically significant odds ratio) between a predictor and an outcome event is not sufficient evidence to conclude that a variable will substantially improve predictive performance [17]. Researchers often suggest that a biomarker is a “powerful predictor” based on weak associations that are highly statistically significant. Statistical significance only indicates that the observed association was most likely not due to random sampling variation. Although the odds ratios for BNP and hs-TnT were highly statistically significant in this example, the magnitude of the increases in relative odds associated with a logarithmic increase in these biomarkers suggest that they will not greatly enhance our ability to discriminate those who did versus did not die within one year of the baseline assessment. The odds ratio for a predictor in a multivariable model needs to be much greater 3.0 to adequately discriminate outcome groups [17]. The odds ratio for the logarithm of the C-reactive protein level was only 1.1 (95% confidence interval 0.98 to 1.24; *P*-value = .11) when added to the baseline clinical prediction model. Thus, adding this biomarker is even less likely to substantially improve our predictive performance.

### 4.2. ROC Curves

**Figure 1:**
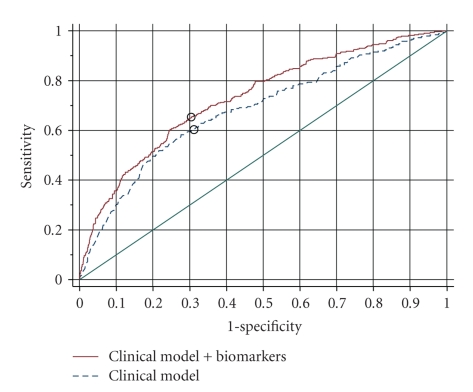
ROC curves. Sensitivity versus 1 minus specificity for discriminating subjects who did or did not die within 1 year based on all possible cut points of predicted probabilities derived from a prediction model that included several baseline clinical assessments (Clinical model) and the same model plus two biomarkers, BNP and hs-TnT. The circles on each curve correspond to the cut point of interest in this example, that is those with a ≥10% predicted probability of dying within 1 year.

Evaluations of prognostic indicators often report receiver operating characteristic (ROC) curves as retrospective measures of discrimination of those that did or did not experience the outcome event during a specified period of time. Adding the BNP and hs-TnT to the baseline model of clinical variables improved the area under the ROC curve from 0.68 (95% confidence interval 0.65 to 0.71) to 0.73 (95% confidence interval 0.70 to 0.76; *P* < .00001) as shown in the Figure 1. However, the ROC curves compare the sensitivity of the prediction models over all, mostly irrelevant, values of specificity. If one is interested in comparing sensitivities and specificities of prediction models, the comparison should focus on the threshold of predicted probability that will be used to define the prognostic groups that will be treated differently. In our example, the cut point for predicted probability of interest is 0.1 (a 10% probability of dying within 1 year) corresponding to the points marked by circles on ROC curves in the Figure 1. The estimated sensitivity of the prediction model with the additional biomarkers was 65.6% (versus 60.4% without the biomarkers) and the specificity was 69.4% (versus 68.8% without the biomarkers). However, estimates of sensitivities and specificities are not sufficient to determine whether or not adding the biomarkers improved our ability to select a group of subjects whose mortality would be predicted to be at least 10%. The PPV in the group of subjects that had an estimated probability of dying of at least 0.1(10%) was 18% after the addition of the biomarkers (versus 16.6% before) and the NPV was 95.1% (versus 94.4% before). The more clinically useful predictive values cannot be gleaned from ROC curves. The improvement in the PPV suggests that subjects selected with the aid of the additional biomarkers would be more likely to die within a year which could be considered an improvement for the purpose of selecting higher risk subjects. However, the increase in the PPV was not substantial or statistically significant (the relative PPV is 1.09 with a 95% confidence interval of 0.9 to 1.3; *P* = .35) [18]. Even if the relative PPV was statistically significantly greater than one, further evaluation would be necessary to decide whether small improvement in predictive value justifies the additional costs of measuring these biomarkers [19].

### 4.3. Reclassification Tables

Reclassification tables are increasingly used by investigators to help compare different methods of risk assessment [20, 21]. A reclassification table for the Val-HeFT example is presented in the Table 1. The addition of BNP and hs-TnT reclassified 342 (15%) of the 2345 subjects who were classified using the other baseline clinical variables as having a predicted probability of dying within 1 year as <10%. Furthermore, 345 (29%) of the 1206 subjects who were previously classified as having a predicted probability of dying within 1 year as ≥10% were reclassified. Judging by the smaller differences between observed and predicted mortality percentages for the column totals compared to the row totals of the Table 1, the model with the two additional predictors appears to be better calibrated to the observed outcomes, that is, the predictive values for each prognostic group. In the prognostic groups with a predicted probability of dying under 10% the observed and predicted mortality percentages were 5.6% and 6.0%, respectively, in the baseline model, compared to 4.9% and 5.0% with the two additional biomarker predictors. In the prognostic groups with a predicted probability of dying ≥10% the observed and predicted mortality percentages were 16.6% and 15.8% in the baseline model, compared to 18.0% and 17.8% with the two additional biomarker predictors. Investigators can report a statistical test to compare the observed and predicted mortality for each model [22]. For the baseline clinical model the J^2^ statistic is 1.02 and the associated *P*-value is .31. For the baseline clinical model plus BNP and hs-TnT the J^2^ statistic is 0.12 and the associated *P*-value is .73. Thus, the observed and predicted mortalities in the defined prognostic groups were not statistically significantly different for either prediction model. The two J^2^ statistics do not indicate whether or not the small improvement in model calibration associated with the addition of the two biomarkers was statistically significant (not likely due to sampling variation) or clinically significant. 

Note the observed percentages dead in the row and column totals for the prognostic groups where the predicted probability was ≥10% are the PPV's cited earlier, 16.6% for model 1 and 18% for model 2. The percentages of observed dead in the row and column totals where the predicted probability was <10% are the complement of the NPV's cited earlier, 100% − 94.4% = 5.6% for model 1 and 100% − 95.1% = 4.9% for model 2. In this example, both prediction models classified about the same number (percentage) of subjects into each prognostic group, but they were not entirely the same subjects. Thus, the reclassification tables provide some interesting insights into the effects of adding the biomarkers on the reclassification of subjects and the calibration of group predictions to the predictive values. 

Reclassification tables can also be retrospectively compiled separately for those who did or did not die within the specified time period to estimate the net reclassification improvement (NRI) [23]. Among the deceased, reclassifications out of the prognostic group with predicted probabilities of dying under 10% would be an improvement whereas reclassifications in the opposite direction would be undesirable. In the example, the classification of 42 out of 331 deceased (12.7%) was better and the classification of 25 deceased subjects (7.6%) was worse when BNP and hs-TnT were added to the prediction model. The net improvement in classification of deceased subjects was 12.7% − 7.6% or 5.1%. Among the survivors, reclassifications out of the prognostic group with predicted probabilities of dying ≥10% would be an improvement whereas reclassifications in the opposite direction would represent worse predictive performance. In the example, the classification of 320 out of 3220 survivors (9.9%) was better and the classification of 300 survivors (9.3%) was worse with the addition of BNP and hs-TnT. The net improvement in classification of survivors was 9.6% − 9.3% or 0.6%. The NRI is the sum of these two net improvements, that is, 5.1% plus 0.6% equal to 5.7%. Statistical tests are available (not reported herein) to determine whether any of these three estimates of improvement in classifying patients into meaningful prognostic groups are significantly different from zero. However, in the case of a dichotomous classification of predicted outcome probabilities the net improvements in each outcome group are equivalent within rounding error to the previously calculated increases in sensitivity (65.6% − 60.4% = 5.2%) and specificity (69.4% − 68.8% = 0.6%). Thus, estimating and testing the NRI would not greatly enhance the evaluation of how much adding the biomarkers to the baseline clinical prediction model improved predictive performance.

## 5. Summary

The research community needs to work in collaboration with clinicians and policymakers to provide more definitive evidence that biomarkers can serve as useful prognostic indicators. To advance this field of study beyond mere identification of biomarkers that provide some prognostic information, clinical investigators will need to

- identify decisions that rely on outcome predictions such as heart replacement, use of costly devices, referral to hospice,
- classify outcome probabilities to define prognostic or risk groups that would generally be treated differently,
- conduct prospective clinical studies of patients encountered in daily practice to estimate and compare predictive values for patients that are placed in prognostic groups that would be treated differently.

Hopefully, studies will be forthcoming to advance this promising use of biomarkers. However, we should not be overly optimistic that biomarkers will substantially improve predictive values [24]. Furthermore, we will have to demonstrate that measuring biomarkers to place patients in different prognostic or risk groups results in a net improvement in patient outcomes that justifies the additional costs.

## Figures and Tables

**Table 1:** Reclassification of Subjects into Prognostic Groups by Adding Two Biomarkers, BNP and hs-TnT, to a Prediction Model Based on Other Baseline Assessments.

Model 1: Baseline Assessments	Model 2: Baseline Assessments + Biomarkers		
	Predicted Probability <10%	Predicted Probability ≥10%	Total
Predicted Probability <10%			
Number of subjects	2003 (85%)*	342 (15%)	2345
Observed Dead	4.4%	12.3%	**5.6%**
Predicted Dead Model 1	5.7%	7.8%	**6.0%**

Predicted Probability ≥10%			
Number of subjects	345 (29%)	861(71%)	1206
Observed Dead	7.2%	20.3%	**16.6%**
Predicted Dead Model 1	13.0%	16.9%	**15.8%**

Total			
Number of subjects	2348 (66%)	1203 (34%)	3551
Observed Dead	**4.9%**	**18.0%**	9.3%
Predicted Dead Model 2	**5.0%**	**17.8%**	

*Percentages in parentheses are calculated across each row.
